# Pseudogout Masquerading as Septic Arthritis in a Patient With Rheumatoid Arthritis

**DOI:** 10.7759/cureus.92545

**Published:** 2025-09-17

**Authors:** Zahra Vaezi, Afshin Amini

**Affiliations:** 1 Internal Medicine, St. Luke's Hospital, Chesterfield, USA

**Keywords:** calcium pyrophosphate deposition disease (cppd), procalcitonin, rheumatoid arthritis, synovial fluid analysis, wrist septic arthritis

## Abstract

Septic arthritis is a medical emergency requiring prompt diagnosis and treatment to prevent joint destruction. However, crystal arthropathies such as calcium pyrophosphate deposition (CPPD) disease can closely mimic infection, particularly in immunosuppressed patients, leading to diagnostic uncertainty and potentially unnecessary antibiotic use. We describe a 67-year-old wheelchair-bound man with seropositive rheumatoid arthritis on methotrexate, hydroxychloroquine, and prednisone who presented with acute left-wrist pain, swelling, and limited range of motion following minor trauma. Laboratory studies showed leukocytosis, elevated CRP, ESR, and mildly increased procalcitonin. Radiographs revealed chondrocalcinosis, and MRI demonstrated pancarpal joint effusion and erosive arthropathy. Given immunosuppression and inflammatory markers, empiric vancomycin was started for presumed septic arthritis. Synovial aspiration yielded scant fluid; microscopy demonstrated numerous rhomboid, weakly positively birefringent crystals consistent with CPPD. Gram stain and culture were negative. Antibiotics were discontinued, and the patient improved rapidly on corticosteroids. This case underscores the importance of synovial crystal analysis in differentiating septic arthritis from pseudogout, particularly in patients with rheumatoid arthritis or immunosuppression. Judicious use of biomarkers (calprotectin, procalcitonin) and early arthrocentesis are essential to avoid overtreatment with antibiotics.

## Introduction

Septic arthritis is a true rheumatologic emergency, with reported mortality rates of 4%-11% and a high risk of rapid, irreversible joint destruction if not promptly treated [1]. The clinical presentation is characterized by acute monoarthritis, pain, erythema, and systemic inflammatory response. However, several non-infectious causes-including crystal arthropathies such as calcium pyrophosphate (CPP) deposition (CPPD) disease-can mimic this presentation [2,3].

CPPD, commonly known as pseudogout, is characterized by the deposition of CPP dihydrate crystals in articular and periarticular tissues. Its prevalence increases with age and is seen in up to 25% of adults over 80 years [4]. The clinical spectrum ranges from asymptomatic chondrocalcinosis to acute CPP crystal arthritis and chronic CPPD-related arthropathy [5]. Importantly, patients with comorbid inflammatory arthritis such as rheumatoid arthritis (RA) may present with acute flares that resemble septic arthritis, leading to diagnostic confusion [6].

Failure to promptly differentiate between septic arthritis and pseudogout has significant therapeutic implications. Overdiagnosis of infection may result in unnecessary hospitalization, intravenous antibiotics, and invasive procedures, whereas missed septic arthritis risks irreversible joint damage [7]. Traditional markers such as ESR and CRP lack specificity, and imaging findings are frequently overlapping [8]. Emerging biomarkers such as synovial calprotectin, apolipoprotein C-I, and serum procalcitonin > 0.5 ng/mL have been proposed as useful adjuncts but are not yet definitive [9,10]. This report describes a case of pseudogout masquerading as septic arthritis in an immunosuppressed RA patient and highlights the role of early arthrocentesis, crystal analysis, and judicious interpretation of biomarkers in guiding management.

## Case presentation

A 67-year-old wheelchair-bound man with seropositive RA presented to the emergency department (ED) with acute-onset pain, swelling, and restricted range of motion of the left wrist. Symptoms developed shortly after a minor trauma involving a yard hoe while gardening. He denied fever, chills, or systemic symptoms. His past medical history included longstanding RA treated with methotrexate, hydroxychloroquine, and low-dose prednisone. He had atrial fibrillation managed with warfarin (international normalized ratio (INR) 2.3), recurrent gout flares, osteoarthritis, spinal stenosis, and multiple joint replacements. Laboratory studies demonstrated mildly elevated leukocyte count and increased CRP and ESR. Serum uric acid and procalcitonin were within normal limits (Table 1).

**Table 1:** Laboratory investigations

Parameter	Day 1 (presentation)	Reference range	Interpretation
WBC count	10.6 × 10³/µL	4.3–10.0 × 10³/µL	Mildly increased
CRP	7.7 mg/dL	<0.5 mg/dL	Increased
ESR	32 mm/hr	<20 mm/hr	Increased
Procalcitonin	0.17 ng/mL	<0.50 ng/mL	Normal
Serum uric acid	5.1 mg/dL	3.5–7.2 mg/dL	Normal

**Figure 1:**
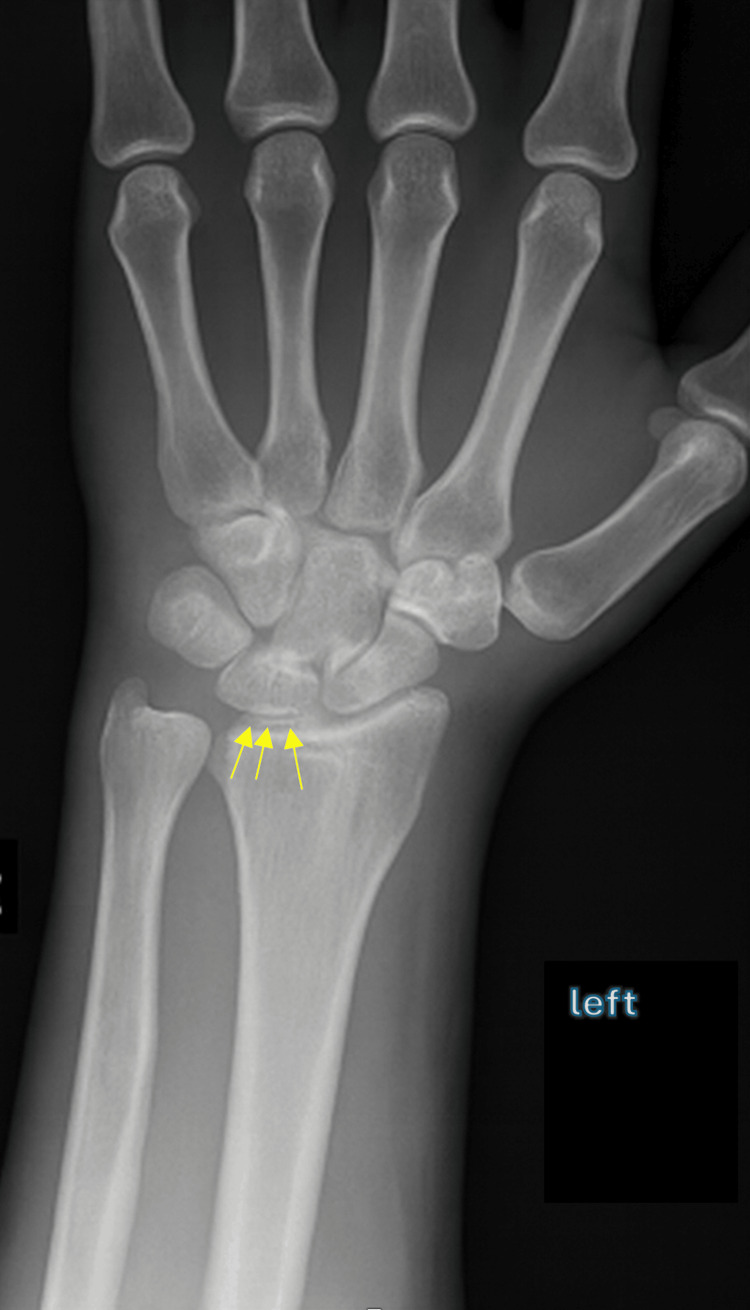
Plain radiograph of the left wrist showing chondrocalcinosis (yellow arrows)

**Figure 2:**
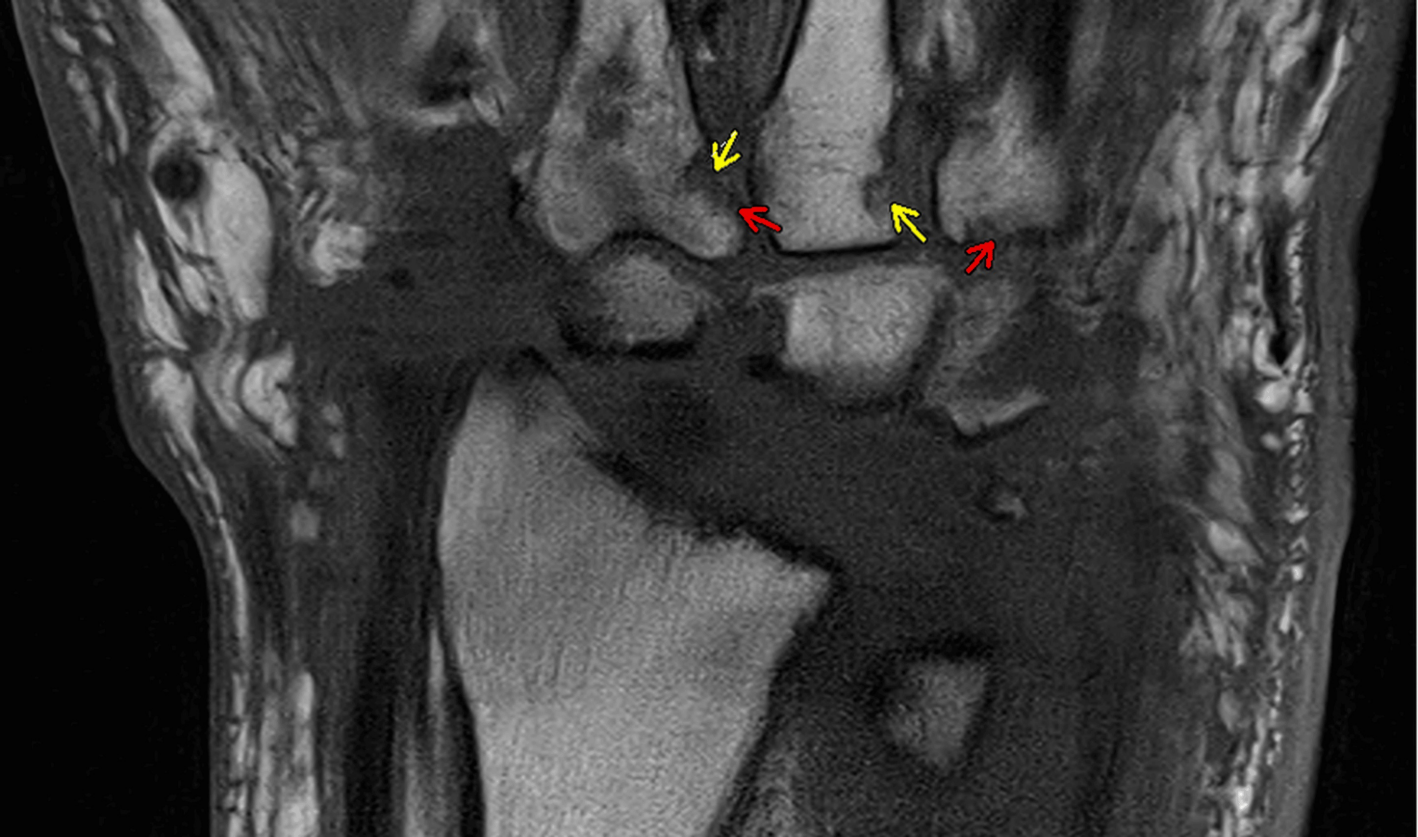
Coronal MRI of the left wrist demonstrating pancarpal joint effusion and synovial thickening (red arrows), along with subchondral cystic changes and erosions (yellow arrows), consistent with chronic inflammatory arthropathy and CPPD flare rather than acute septic arthritis CPPD: calcium pyrophosphate deposition

In the ED, left-hand radiographs demonstrated chondrocalcinosis (Figure 1). Based on the erythema, swelling, and tenderness of the left wrist, the initial working diagnosis was cellulitis, and empiric intravenous vancomycin was started. MRI of the wrist was subsequently performed, demonstrating pancarpal joint effusion, osseous erosions, and subcortical cysts (Figure 2), raising suspicion for septic arthritis.

**Figure 3:**
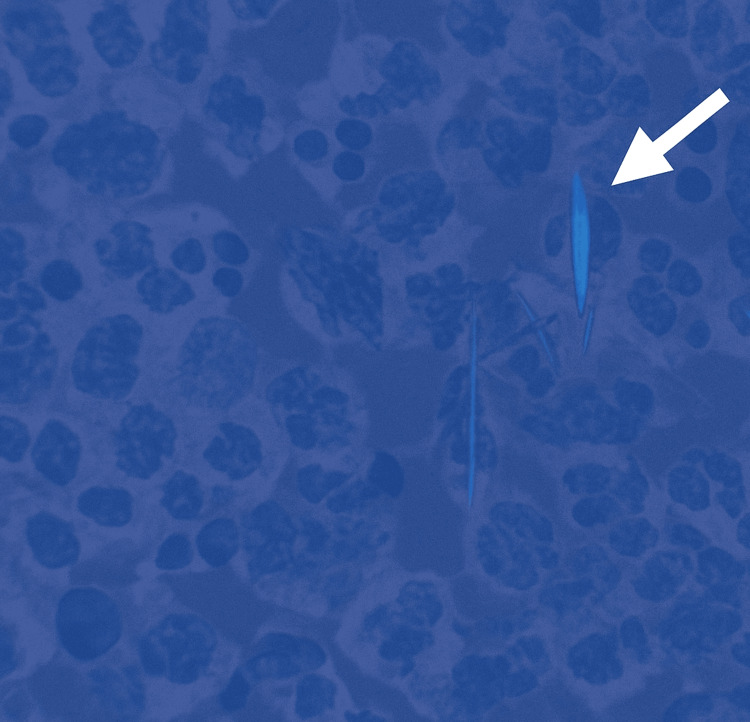
Polarized light microscopy image showing rhomboid, weakly positively birefringent calcium pyrophosphate dihydrate (CPPD) crystals (white arrow)

In response, piperacillin-tazobactam (Zosyn) was added to vancomycin to broaden antimicrobial coverage. Arthrocentesis was then performed after the initiation of empiric antibiotics, yielding a small volume of synovial fluid insufficient for cell count. Polarized light microscopy revealed rhomboid, weakly positively birefringent crystals consistent with CPPD (Figure 3).

Gram stain was negative, and bacterial cultures remained sterile. After infectious disease consultation, antibiotics were discontinued. He was treated with intravenous methylprednisolone followed by an oral prednisone taper, leading to the rapid resolution of pain and swelling.

## Discussion

This case highlights the nuanced diagnostic challenge of acute monoarthritis in an immunosuppressed host. Septic arthritis is potentially devastating, with a reported mortality of up to 11% and permanent joint damage in more than 30% of cases if diagnosis and treatment are delayed [1]. CPPD, by contrast, is common in the elderly, with radiographic chondrocalcinosis seen in up to 15% of people over age 65 [2,3]. Its presentation as acute pseudogout can mimic infection so closely that empiric antibiotics are often started (Table 2).

**Table 2:** Clinical, laboratory, and imaging features that aid in differentiating septic arthritis from pseudogout. Table created by the authors (table credits: Vaezi & Amini) based on data synthesized from References [1–9] CPPD: calcium pyrophosphate deposition; OA: osteoarthritis; RA: rheumatoid arthritis

Category	Septic arthritis	Pseudogout (CPPD)	Pearl/clue
Onset & course	Hyperacute, rapid progression	Acute/subacute, recurrent flares	Explosive onset favors infection [1]
Systemic symptoms	Fever, chills, malaise common	Usually afebrile	Absence of fever does not exclude infection [1]
Risk factors	Prosthetic joint, diabetes, bacteremia	OA, metabolic disease	RA risk factor for both [2]
Synovial WBC	>50,000 (neutrophilic)	2,000-50,000 (neutrophilic)	>100,000 strongly favors infection [1]
Crystals	Absent (except coexistence)	Rhomboid crystals present	Crystals do not exclude infection [4-6]
Biomarkers	High calprotectin, ApoC1 [7,8]	Lower calprotectin	Useful rapid diagnostic tool [7]
Procalcitonin	>0.5 ng/mL	Normal/mild elevation	Helpful rule-in [9]

Coexistence of septic arthritis with crystal arthropathy complicates the picture further. Multiple case series and reports document simultaneous bacterial infection and CPPD in the same joint, particularly in elderly or immunocompromised patients [4-6]. For this reason, crystal identification should not preclude Gram stain and culture. Our patient’s negative culture and rapid corticosteroid response argued strongly for pseudogout flare rather than infection. Biomarker analysis can be a useful adjunct. Synovial fluid calprotectin has demonstrated high diagnostic accuracy, with sensitivities above 90% and specificities near 85% for septic arthritis compared to crystal-induced arthritis [7]. Apolipoprotein C1 levels are also elevated in septic arthritis compared with RA, though they do not reliably distinguish CPPD [8]. Procalcitonin > 0.5 ng/mL is a helpful rule-in marker for bacterial infection but has limited sensitivity; normal values do not fully exclude septic arthritis [9]. In this case, procalcitonin was only mildly elevated, supporting the de-escalation of antibiotics. Imaging, while non-specific, plays a complementary role. MRI can reveal effusion, synovial enhancement, or bone marrow edema but cannot reliably distinguish infection from inflammation. Findings such as periarticular abscess or cortical bone destruction should heighten suspicion for septic arthritis. It is also notable that immune triggers such as vaccination may precipitate CPPD flares, an increasingly recognized phenomenon post-COVID-19 [10]. Our patient had no recent vaccination, but this association underscores the need to carefully explore potential triggers during history-taking. Therapeutically, a rapid clinical response to corticosteroids or non-steroidal anti-inflammatory drugs (NSAIDs) is characteristic of pseudogout. This case underscores the importance of multidisciplinary management involving rheumatology and infectious disease, early arthrocentesis prior to antibiotic initiation, and careful interpretation of biomarkers to avoid both undertreatment and overtreatment. Prompt recognition and targeted therapy can lead to complete symptom resolution and prevent unnecessary antimicrobial exposure.

## Conclusions

CPPD (pseudogout) is an important mimic of septic arthritis, particularly in patients with RA and those who are immunosuppressed. This case underscores that the presence of acute monoarthritis with elevated inflammatory markers should not immediately trigger prolonged empiric antibiotics without confirmatory diagnostic data. Early joint aspiration with crystal analysis, Gram stain, and culture remains the gold standard for differentiation. Adjunctive biomarkers such as calprotectin and procalcitonin may support clinical decision-making but must be interpreted in the context of clinical findings and imaging. Rapid recognition of CPPD flare allows for timely initiation of targeted anti-inflammatory therapy (NSAIDs, colchicine, or corticosteroids), which can result in dramatic clinical improvement, while avoiding the risks of unnecessary antibiotics, hospital stay, and antimicrobial resistance. Multidisciplinary collaboration between rheumatology, infectious disease, and orthopedics is crucial for optimizing outcomes and preventing long-term joint damage.
